# Two Cases of Appendiceal Intussusception: A Rare Diagnostic Pitfall in Colonoscopy

**DOI:** 10.1155/2011/198984

**Published:** 2011-04-12

**Authors:** Hassan Seddik, Monsef Rabhi

**Affiliations:** ^1^Department of Gastroenterology, Mohammed V Military Teaching Hospital, Rabat, Morocco; ^2^Department of Internal Medicine, Mohammed V Military Teaching Hospital, Rabat, Morocco

## Abstract

Partially or completely invaginated appendix mistaken for a polyp during colonoscopy and leading to intussusception is a rare situation. This paper describes our experience with two cases of appendiceal intussusception. In the first case, there was no underlying ileocecal abnormality, and, in the second case, histologic examination of the resected appendix and cecum revealed widespread foci of angiodysplasia, and this was thought to be the basis for the intussusception. The authors present reviews of the literature concerning clinical features and associated conditions and emphasize that failure to recognize this condition may result in unexpected complications such as consequent peritonitis in case of endoscopic removal.

## 1. Introduction

Intussusception of the appendix is an extremely rare condition. It affects all ages but is usually associated with males in the first decade [1]. Despite its rarity, endoscopists must consider appendiceal intussusception in the differential diagnosis when a “polyp” is seen on colonoscopy in order to avoid a consequent peritonitis in case of endoscopic removal. We report here two cases: the first was fortuitously discovered on colonoscopy, and the second was responsible for lower- gastrointestinal-tract bleeding. We insist on morphological characteristics as to permit an early recognition and treatment of this condition.

## 2. Case Report

### 2.1. Case 1

**Figure 1:**
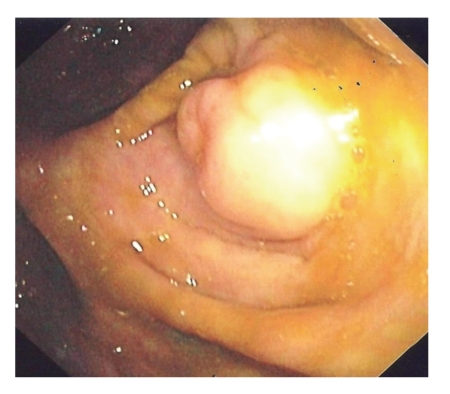
Sessile, dimpled 20 mm polypoid mass in the cecum. Colonoscopic image.

A 48-year-old man with several years history of abdominal pain and transit disturbances was admitted to our hospital for further investigations. Physical examination and laboratory data were within normal limits. Colonoscopy revealed a sessile, dimpled 20 mm cecal polyp (Figure 1) covered with macroscopically normal colonic mucosa. The appendiceal orifice in the cecum was not seen. Biopsies were performed, and, 12 hours after, the patient experienced fever and shivering. Clinical examination of the abdomen and plain abdominal roentgenogram were normal. Complete recovery was obtained after broad spectrum antibiotherapy. On histology, the polyp was found to be normal inverted appendiceal wall. Abdominal ultrasound and CT scan showed a partially invaginated appendix into the cecal cavity without any evidence of tumoral process. Regular ultrasound examinations during two years showed an unchanged aspect of the inverted appendix

### 2.2. Case 2

**Figure 2:**
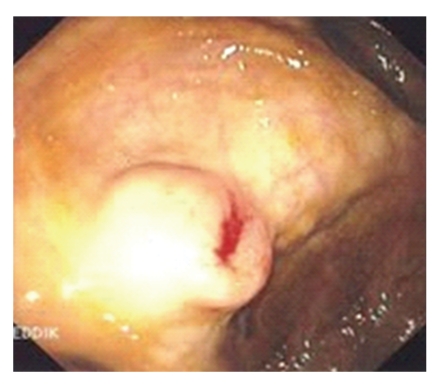
Actively bleeding, sessile, polypoid mass in the cecum. Colonoscopic image.

A 65-year-old man was admitted to our hospital with a six-month history of melena with transfusion requirement. Hemogram showed hypochromic microcytic anemia* (hemoglobin 8* g* dL-1*). Gastroscopy was normal but colonoscopy showed an actively bleeding, sessile, cecal polyp located in the usual site of the appendiceal orifice (Figure 2). There were three angiodysplastic lesions in the rest of the cecum, without active bleeding. An abdominal CT scan showed an invaginated appendix. The exploration of the small bowel using video capsule endoscopy did not reveal any other source of bleeding. During surgery, the appendix was found to be partially invaginated into the cecal cavity, with bleeding through the appendiceal orifice. Ileocecal resection was performed. On pathological examination, the resected specimen was found to contain angiodysplastic foci without any malignant lesions. The patient's recovery from surgery was uneventful. No recurrence of the lower-gastrointestinal-tract bleeding was observed during followup, and anemia was corrected after oral iron therapy.

## 3. Discussion

Appendiceal inversion was first described in 1858 [2]. It is an uncommon condition with an incidence rate of 0.01% in a large autopsy series [3]. Pathophysiology remains unclear but several etiologies have been described [4], anatomical variations of the appendix, such as fetal type cecum, a wide appendicular lumen, and a thin, mobile appendix; or pathological conditions such as tumours (polyps, mucinous cystadenoma, adenocarcinoma, carcinoid tumor, and GIST), endometriosis, parasitism, cystic fibrosis, abnormal appendicular peristaltism, fecaliths, and foreign bodies [1, 4–7]. Intussusception of the resultant appendiceal stump after inversion-ligation appendicectomy has been described [4]. However, appendiceal intussusception may occur without any underlying abnormality. Case 2 is interesting because appendiceal intussusception associated with angiodysplasia of the appendix has never been reported to our knowledge and could be a cause-effect relationship. Patients tend to present with symptoms of abdominal pain, small bowel obstruction, and rectal bleeding; the clinical presentation may also mimic acute appendicitis [1, 4]. A few cases in asymptomatic patients have been incidentally diagnosed by barium enema, colonoscopy, CT colonography, or endoscopic ultrasound. Careful endoscopic examination, identifying the appendiceal orifice, should be required in the case of cecal polyp. Endoscopic removal of this lesion is associated with a high risk of peritonitis [7]. In case 1, simple biopsies have provoked bacterial infection. It is therefore advisable to perform gross examination of all caecal polyps after colonoscopic removal. A recent advance is the use of through-the-scope miniprobe catheter endoscopic ultrasound to evaluate abnormal findings of the appendix identified by colonoscopy and allow selection of those in need of surgical management [5]. Preligation with Endoloop and postpolypectomy ligation technique using the Anchor clip may minimize the risk of postpolypectomy hemorrhage [8–10].
